# Diabetic Neuropathy: A Clinical and Neuropathological Study of 107 Patients

**DOI:** 10.1155/2010/140379

**Published:** 2010-06-06

**Authors:** David S. Younger

**Affiliations:** Department of Neurology, New York University School of Medicine, New York, NY 10016, USA

## Abstract

One hundred seven patients were retrospectively studied from 1992 to 2002 with diabetic neuropathy that underwent peripheral nerve biopsy. Nerve biopsy revealed the underlying histopathology, including cell and humoral-mediated immunological lesions in the majority of patients. When combined with clinical and laboratory studies, nerve biopsy has the potential to assist in the selection of patients who may benefit from immunomodulatory therapy.

## 1. Introduction

There are few modern series of diabetic neuropathy confirmed by nerve tissue obtained at biopsy or postmortem examination available to allow clinicopathological correlation of the different neuropathic syndromes. In 1996, Younger et al. [1] reported clinicopathological and immunohistochemical findings in 20 patients with heterogeneous forms of diabetic neuropathy. The present report continues that series to a total of 107 patients who underwent detailed clinicopathological assessment.

## 2. Methods

One hundred seven consecutive patients with diabetes mellitus and peripheral neuropathy comprising distal symmetrical polyneuropathy (DSPN), proximal diabetic neuropathy (PDN) hereafter referred to as diabetic lumbosacral radiculoplexus neuropathy (DLRPN) [2, 3], and mononeuritis multiplex (MNM) at the New York Presbyterian Hospital and New York University Langone Medical Center were identified from 1992 to 2002. Causes of neuropathy other than diabetes mellitus were carefully excluded by appropriate investigations. Peripheral neuropathy was established by standard nerve conduction studies (NCSs) and needle electromyography (EMG). 

Patients with DSPN presented with distal leg sensory and later motor involvement manifested variably by pain, paresthesia, limb weakness, gait disturbance, imbalance, and hyporeflexia in the legs. Patients with DLRPN presented with pelvi-femoral pain followed by weakness beginning focally and unilaterally, affecting the leg and thigh, progressing to involve the lower leg with spread to the contralateral limb, combined with sensory loss and concomitant weight loss often in excess of 10 pounds. MNM presented with a stepwise pattern of motor and sensory loss in the distribution of individual peripheral nerves. 

Patients were referred for peripheral nerve biopsy after diagnosis of symptomatic peripheral neuropathy to ascertain the histopathological basis of neuropathy, not based on specific clinical or electrophysiological features and performed along the sural nerve above the ankle according to standard technique [1, 4] after obtaining informed consent. All biopsy specimens were analyzed by the Department of Pathology, Columbia University. The sural nerve was chosen for biopsy because it is the most widely studied nerve histopathologically. In all but one patient with MNM, the sural nerve was involved clinically and electrophysiologically. The status of control of diabetes mellitus at the time of nerve biopsy was comparable for all patients. 

The neuropathology was classified as primarily axonopathy and myelinopathy after analysis of semithin epoxy sections and teased nerve fiber preparations. The severity was categorized as mild, moderate, or severe based upon the degree of myelinated fiber degeneration and loss in transverse paraffin and epoxy sections. Mononuclear inflammatory cell infiltration in or around epineurial vessel walls, respectively, defined micovasculitis (MV) and perivasculitis (PV) [1]. Necrotizing arteritis (NA) was diagnosed by active or healed lesions in arteriae nervorum [5]. The frequency of MV, PV, and NA was not subcategorized according to the clinical presentation of neuropathy.

## 3. Results

The clinical and neuropathological findings are summarized in Table 1. Two patients had juvenile-onset diabetes mellitus, and the remainder had types 1 and 2 in equal ratio. One patient had MNM, and the remainder had DSPN and DLRPN in a 2 : 1 ratio. Five patients (4%) had minor wound infection at the incision site that responded to antibiotics and 5 (4%) had short-lasting postoperative causalgia. One patient with DSPN had normal nerve pathology. 

**Figure 1:**
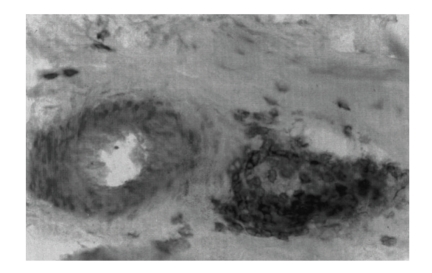
Biopsy of sural nerve in cross section in a patient with diabetic lumbosacral radiculoplexus neuropathy (DLRPN) demonstrates focal intense mononuclear inflammatory cells that surround and invade the wall of a small epineurial blood vessel on the right side of the photomicrograph indicative of microvasculitis (MV) (Cryosection, Immunoperoxidase, 400x).

**Figure 2:**
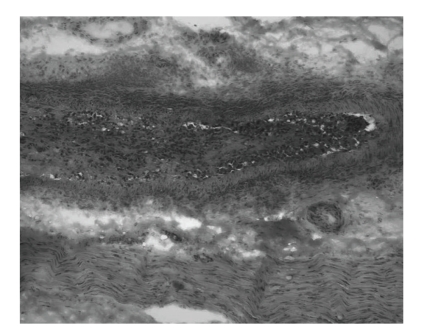
Biopsy of sural nerve in longitudinal section demonstrates features of necrotizing arteritis (NA) in a patient with diabetic lumbosacral radiculoplexus neuropathy (DLRPN). Numerous inflammatory cells surround a small epineurial artery. The lumen contains reactive connective tissue with recanalization, thrombus formation, and vessel wall necrosis. An adjacent nerve fascicle has a marked loss of myelinated fibers (Cryosection, H&E, 200x).

The severity of neuropathy was mild in 17%, moderate in 50%, and severe in 33%. Two-thirds of nerves were deemed primary axonopathy, and one-third primary myelinopathy. Altogether, 3% and 23% of nerves, respectively, revealed MV (Figure 1) and PV. Immunofluorescence showed C3 and C5b-9 membrane attack complex deposits in the walls of endoneurial microvessels in two-thirds of nerves. Necrotizing arteritis (Figure 2), detected in nerve biopsy tissue of two patients with DSPN and one DLRPN, was absent in postmortem tissue of the latter case in which femoral, sciatic nerve, and lumbar plexus [6] showed PV of the epineurium, perineurium, and endoneurium.

## 4. Discussion

The frequency of MV (3%) and PV (23%) was reduced compared to 60% and 40%, respectively, when immunoperoxidase immunoflourescent staining was employed [1]. Necrotizing arteritis was not verified in the postmortem studied case. The 3% frequency of MV in our cohort compared favorably with 6% reported in DLRPN [2]. Mononuclear cell infiltration was first noted in the peripheral nerves of patients with diabetes mellitus obtained at postmortem and in amputated limbs [7]. Later, PV [2, 8], MV [2, 9], and NA [2, 10] were noted in sural [2, 8], superficial peroneal sensory [2, 9], and femoral intermedius nerve biopsies [9, 10]. The infiltrating mononuclear inflammatory cells proved to be activated CD8+ T cells expressing pathogenic cytokines and major histocompatibility class II antigen [1], in association with activated complement. It was not possible to correlate the frequency and severity of peripheral nerve inflammation with the clinical forms of diabetic neuropathy in the present cohort since the nerves demonstrating MV and NA were very few in number, and those showing PV were not further stratified into subgroups of severity. Further, the present study did not ultrastructurally examine diabetic endoneurial microvessels that have previously shown reduplication of basement membranes and pericyte degeneration, which can lead to alterations in the size or the vessel lumen, thickening of vessel walls, alterations in the blood-nerve barrier, and endoneurial microenvironment [11]. 

Axonopathy was the predominant pathology overall in the present cohort, however a third of nerves demonstrated demyelination. The latter has historically been attributed to axonal degeneration as the primary ischemic event. Segmental demyelination and remyelination was observed in 23% of sural nerve biopsies due to primary axonal degeneration, and in 12/33 sural nerves in DLRPN [2], with vascular inflammatory cell collections. Anecdotal reports of patients with chronic inflammatory demyelinating polyneuropathy (CIDP) and diabetes mellitus [12] support primary autoimmune demyelinating nerve disease, however we were unable to retrospectively define cases of CIDP according to strict criteria available during the study period [13]. 

The finding of necrotizing arteritis and microvasculitis in the peripheral nerve biopsies of several patients with heterogeneous forms of diabetic neuropathy lends further support to ischemic axonopathy without invoking a concomitant systemic vasculitic process. Patchy fiber loss of upper sciatic nerve with severe length-dependent distal fiber loss and axonopathy [12] was noted in postmortem tissue of two diabetics with distal symmetrical sensory polyneuropathy and reminiscent of necrotizing angiopathic neuropathy (NAN) [14]. Notwithstanding, it remains enigmatic that neither systemic nor widespread peripheral nerve vasculitis was detected in postmortem tissue from the patients with NA in sural nerve biopsy, or in the postulated cases of diabetic NAN [12]; more likely, MV and severe PV contribute more to epineurial ischemia than does NA. 

Although this study did not examine treatment choices made for individual patients or subgroups of the cohort based upon nerve biopsy results, the present findings of sural nerve inflammation and demyelination may be a clue to an underlying autoimmune etiopathogenesis in some affected patients. Future studies should address the elucidation of prospective clinical and laboratory criteria to select patients for open peripheral nerve biopsy in view of the availability of epidermal nerve fiber (ENF) studies by punch skin biops [15]. The latter is less invasive and instead measures ENF density and histology but does not sample large myelinated nerve fiber bundles or epineurial blood vessels which may be clue to systemic or nonsystemic peripheral nerve vasculitis [16].

## Figures and Tables

**Table 1:** Clinical Characteristics of 107 Patients and Diabetic Neuropathy.

Study Cohort	Number	Percentages
Women	36	34
Men	71	66
Mean Age 64.7 years		
(Range 31–95 years)		

Clinical Neuropathic Syndrome		
MNM	1	1
DLRPN	35	33
DSPN	71	66

Histological Severity of Neuropathy		
Normal	1	1
Mild	17	16
Moderate	54	50
Severe	35	33

Teased Fiber/Semithin Section Analysis		
Normal	1	1
Axonopathy	45	65
Myelinopathy	23	35

Cellular Response		
Perivasculitis	26	23
Microvasculitis	3	3
Necrotizing arteritis	3	3

Complement Immunofluorescence		
C3 deposition	70	67
C5b-9	65	62

Other Findings		
Onion bulb formation	10	9
Epineurial vascular thrombosis	5	5
